# Role of the Vasopressin/Apelin Balance and Potential Use of Metabolically Stable Apelin Analogs in Water Metabolism Disorders

**DOI:** 10.3389/fendo.2017.00120

**Published:** 2017-05-31

**Authors:** Adrien Flahault, Pierre Couvineau, Rodrigo Alvear-Perez, Xavier Iturrioz, Catherine Llorens-Cortes

**Affiliations:** ^1^Laboratory of Central Neuropeptides in the Regulation of Body Fluid Homeostasis and Cardiovascular Functions, Center for Interdisciplinary Research in Biology (CIRB), INSERM, U1050/CNRS, UMR 7241, College de France, Paris, France

**Keywords:** apelin, vasopressin, apelin receptor, metabolically stable apelin analogs, G protein-coupled receptor, diuresis, osmolality, apelin water metabolism disorders

## Abstract

Apelin, a (neuro)vasoactive peptide, plays a prominent role in controlling body fluid homeostasis and cardiovascular functions. In animal models, experimental data demonstrate that intracerebroventricular injection of apelin into lactating rats inhibits the phasic electrical activity of arginine vasopressin (AVP) neurons, reduces plasma AVP levels, and increases aqueous diuresis. In the kidney, apelin increases diuresis by increasing the renal microcirculation and by counteracting the antidiuretic effect of AVP at the tubular level. Moreover, after water deprivation or salt loading, in humans and in rodents, AVP and apelin are conversely regulated to facilitate systemic AVP release and to avoid additional water loss from the kidney. Furthermore, apelin and vasopressin secretion are significantly altered in various water metabolism disorders including hyponatremia and polyuria-polydipsia syndrome. Since the *in vivo* half-life of apelin is in the minute range, metabolically stable apelin analogs were developed. The efficacy of these lead compounds for decreasing AVP release and increasing both renal blood flow and diuresis, make them promising candidates for the treatment of water retention and/or hyponatremic disorders.

## Discovery

The apelin story began in 1993 with the cloning of the cDNA for the APJ receptor putative receptor protein related to the angiotensin II receptor type 1 (AT1) from a human genomic library (1). This receptor is a type A G protein-coupled receptor (GPCR) that shares 31% of amino acid sequence identity with the sequence of the human AT1 receptor. The human and the rat APJ receptors are 380 and 377 amino acids long, respectively. Studies have been performed to see if APJ receptor could bind angiotensin peptides due to its close homology with AT1 receptor. Binding experiments with angiotensin peptides were performed on Chinese Hamster Ovary (CHO) cells stably expressing the rat APJ receptor fused to enhanced Green Fluorescent Protein (EGFP). No specific binding for angiotensin II (Ang-II), angiotensin III (Ang-III), or angiotensin IV (Ang-IV) was observed in these cells. Moreover, stimulation of the rat APJ receptor by Ang-II or Ang-III at a concentration of 10^−7^ M did not alter forskolin (FSK)-induced cAMP production, showing no activation of the rat APJ receptor by angiotensin peptides (2). These results showed that APJ receptor was not related to angiotensin peptides and remained an orphan GPCR for which the endogenous ligand had to be discovered.

In 1998, Tatemoto et al. isolated the endogenous ligand of the orphan APJ receptor from bovine stomach extracts. They used a Cytosensor microphysiometer to detect the metabolic activation of cells expressing the human APJ receptor through measurement of the extracellular acidification rate (3). This 36-amino acid peptide was called apelin for *AP*J *E*ndogenous *LI*ga*N*d. Following the discovery of apelin, the APJ receptor was renamed the apelin receptor (ApelinR).

## Gene Encoding/Processing for Apelin and the ApelinR

**Figure 1:**
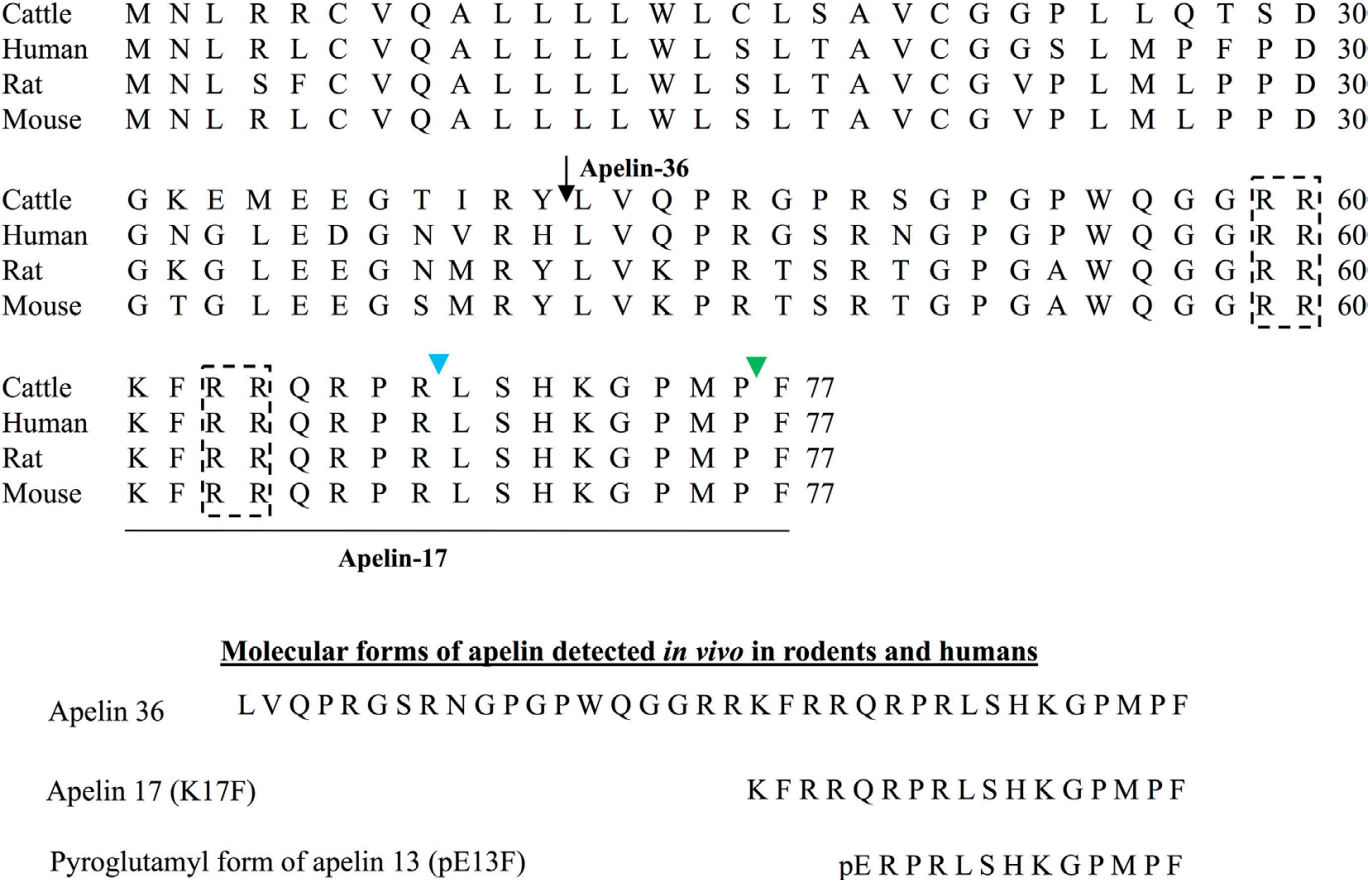
Amino acid sequences of the apelin precursor, preproapelin, in cattle, humans, rats, and mice and the molecular forms of apelin detected *in vivo*. Alignment of preprapelin sequences in cattle, humans, rats, and mice. The arrow indicates the beginning of the sequence of apelin-36 and the apelin-17 (K17F) sequence, strictly conserved in mammals, is underlined. The black dashed boxes show the dibasic doublets that could be recognized by prohormone convertases, potentially involved in preproapelin maturation. The green arrow shows the cleavage site by ACE-2 (EC 3.4.17.23). The blue arrow shows the cleavage site by Neprilysin (EC 3.4.24.11). The various molecular forms of apelin detected *in vivo* in mammals: apelin-36, apelin-17, and the pyroglutamyl form of apelin-13. Figure adapted from Ref. (11) with permission from the copyright holders.

Apelin is generated from a 77-amino acid precursor, preproapelin (Figure 1). The gene encoding preproapelin is located on chromosome X at locus Xq25-26.1 in human, Xq35 in rat and XA3.2 in mouse (4). The human gene contains three exons, with the coding region spanning exons 1 and 2. The 3′ untranslated region also spans two exons (2 and 3) (4). This may account for the presence of two different sizes of transcripts (≈3 and ≈3.6 kb) in various tissues (4, 5). Alignment of preproapelin amino acid sequences from cattle, humans, rats, and mice shows strict conservation of the C-terminal 17 amino acids (amino acids 61–77 of preproapelin sequence), known as apelin-17 or K17F (Figure 1). *In vivo*, various molecular forms of apelin are present, differing only in length (either 36, 17, or 13 amino acids at the C-terminal part of the precursor) (6–10) (Figure 1).

The presence of pairs of basic residues within the cattle, human, rat, and mouse preproapelin sequences suggests that prohormone convertases could be responsible for the processing of the precursor to give birth to K17F and pE13F (pyroglutamyl form of apelin-13: amino acids 65–77 of the preproapelin sequence). More recently, it has been shown *in vitro* that proprotein convertase subtilisin/kexin 3 (also named furin) may cleave proapelin (amino acids 23–77 of preproapelin sequence) directly into apelin 13 without generating longer isoforms (12). For apelin-36 (amino acids 42–77 of the preproapelin sequence) because of the absence of dibasic motifs upstream the apelin-36 cleavage site, the maturation mechanism remains to be defined. Apelin-36 predominates in rat lung, testis, uterus, and in bovine colostrums, whereas both apelin-36 and pE13F have been detected in the rat mammary gland (6, 8). In rat brain as well as in rat and human plasma, the predominant forms of apelin are pE13F and K17F, whereas the concentration of apelin-36 is much lower (9, 10).

The gene encoding for the ApelinR is intronless in human and rodents and it is located on chromosome 11q12 in human (1), 2E1 in mouse, and 3q24 in rat (2, 5, 6). The human and the rat ApelinRs are 380 and 377 amino acids long, respectively. The ApelinR amino acid sequence is conserved across species, with more than 90% of homology between human and rodents, and up to 50% of homology with other non-mammalian species such as zebrafish or frog (2, 5, 6, 13). In contrast, Ang-II and apelin-13 do not show much homology; in fact, Ang-II (amino acid sequence: D-R-V-Y-I-H-P-F) only has in common with pE13F (pE-R-P-R-L-S-H-K-G-P-M-P-F) its two C-terminal amino acid residues (P–F). This explains why both peptides are cleaved by the carboxypeptidase, angiotensin-converting enzyme type-2 (ACE-2, EC 3.4.17.23) (14, 15).

## Metabolism of Apelin Peptides and Pharmacological Characterization of the ApelinR

ACE-2 removes the C-terminal phenylalanine of either apelin-36 K17F or pE13F (14, 15). Finally, it has been recently shown that neutral endopeptidase 24.11 or Neprilysin (EC 3.4.24.11) hydrolyzes the scissile peptide-bond Arg^8^-Leu^9^ of K17F and Arg^4^-Leu^5^ of pE13F leading to two truncated inactive peptides (16).

Apelin peptides exhibit subnanomolar affinities for the ApelinR (17, 18). Alascan studies of pE13F showed that Arg^2^, Arg^4^, Leu^5^ of the RPRL motif of pE13F are key elements for ApelinR binding together with Ser^6^, Lys^8^, and Met^11^ but with a lesser extent (17). Later structure–function studies by molecular modeling and site-directed mutagenesis demonstrated that Arg^2^, Arg^4^, and Lys^8^ of pE13F interact with residues located at the top of the receptor, Glu^172^, Asp^282^, and Asp^92^, respectively (19).

Several studies have explored the signaling pathways activated by the apelin/ApelinR system. Apelin-36, K17F, apelin-13 (Q13F), and pE13F have been shown to have a similar potency (in the subnanomolar range) to inhibit FSK-induced cAMP production in CHO cells expressing the rat ApelinR and human embryonic kidney (HEK) cells expressing the human ApelinR (2, 7, 17, 20). Hosoya et al. (6) showed that Pertussis toxin was able to inhibit apelin-36 and pE13F responses demonstrating that ApelinR was coupled to Gα_i_. This was confirmed by Masri et al. who showed that ApelinR is preferentially coupled to Gα_i1_ and Gα_i2_ protein, which leads to the inhibition of adenylate cyclase and ERK1/2 phosphorylation but was not coupled to Gα_i3_ protein (21, 22).

Apelin-36, K17F, and pE13F have also been shown to increase [Ca^2+^]_i_ mobilization in Ntera 2 human teratocarcinoma (NT2N) cells, which differentiate into postmitotic neurons following retinoic acid stimulation and also in cells derived from basophils (RBL-2H3) or HEK 293 cells stably expressing human ApelinR (17, 23–25). However, the mechanisms underlying this production of calcium remain unknown. Moreover, Hus-Citharel et al. (26) showed that K17F decreases Ang-II-induced [Ca^2+^]_i_ mobilization in glomerular arterioles in a nitric oxide (NO)-dependent manner. Interestingly, several studies have shown that the stimulation of ApelinR by apelin induces vasodilation and modulates vascular tone through NO production (22–24, 27, 28). Furthermore, several kinases have been reported to be activated following apelin-induced ApelinR activation. Among these, the extracellular-regulated kinases (ERKs) are phosphorylated in CHO cells stably expressing the mouse ApelinR in a Gα_i_-protein-dependent, protein kinase C (PKC)-dependent, and Ras-independent manner (21, 29). Apelin also stimulates phosphorylation of S6 ribosomal protein kinase (p70S6K) in human umbilical vein endothelial cells (HUVECs) and in CHO cells expressing the mouse ApelinR *via* two distinct signaling pathways—the phosphatidylinositol 3-kinase (PI3K)/Akt pathway and the ERK1/2 pathway (29, 30). Later on, D’Aniello et al. (31) have shown that apelin induces phosphorylation of p70S6 kinase in murine embryonic stem cells *via* an ERK1/2-dependent pathway.

Finally, as for most GPCRs, rat and human ApelinRs internalize upon the action of agonist ligands such as apelin-36, K17F, and pE13F (17, 18, 20, 25, 32). However, K17F is 30 times more potent in inducing rat ApelinR internalization when compared to pE13F (33). Deletion of the C-terminal Phe of K17F (K16P) or its substitution by an alanine (K17A) strongly decreases the ability of the peptide to trigger ApelinR internalization without effect on its affinity for the ApelinR or its ability to activate Gαi-coupling (18, 20). This suggested that the C-terminal Phe of K17F or pE13F is a key residue to trigger ApelinR internalization (20). These results were supported by structure–function studies combining molecular modeling and site-directed mutagenesis of the ApelinR.

**Figure 2:**
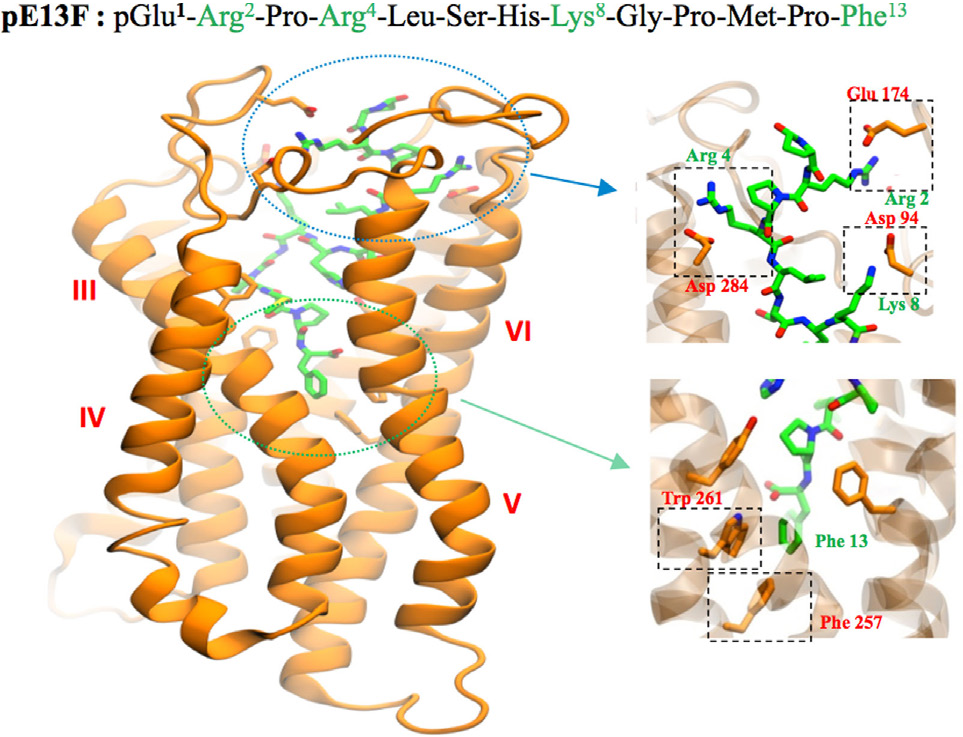
Representation of the human ApelinR three-dimensional model complexed with pE13F. The peptide backbone of the ApelinR is in orange while pE13F is in green. The blue dashed circle shows a detailed view of the binding site of pE13F with interactions (square dashed boxes) between basics residues of pE13F (Arg^2^, Arg^4^, and Lys^8^, in green) and acidic residues of ApelinR (Asp^94^, Glu^174^, and Asp^284^, in orange). The green dashed circle shows a detailed view of the hydrophobic cavity within ApelinR in which the C-terminal Phe of pE13F (Phe^13^, in green) interacts with aromatic residues of ApelinR (Phe^257^ and Trp^261^, in orange). Figure adapted from Ref. (19) with permission from the copyright holders.

Docking of K17F into the three homology three-dimensional (3D) model of the ApelinR using the validated cholecystokinin receptor-1 3D model as a template revealed the presence at the bottom of the binding site of a hydrophobic cavity in which the C-terminal Phe of pE13F was embedded by Trp^152^ in TMIV and Trp^259^ and Phe^255^ in TMVI (18) (Figure 2). Using site-directed mutagenesis, Phe^255^ and Trp^259^ were shown to be crucial for ApelinR internalization without playing a role in apelin binding or Gα_i_-protein coupling, by interacting with the C-terminal Phe of pE13F.

All these data indicate functional dissociation between ApelinR G_i_-coupling and receptor internalization. This implies that the ApelinR exists in different active conformations depending on the ligand fitting into the binding site leading to the activation of different signaling pathways and subsequent different biological effects (18). Altogether, this suggests that ApelinR may exhibit “functional selectivity” or “biased signaling” by, on one hand, coupling with G protein and on the other hand by recruiting β-arrestins 1 and 2 (34). This hypothesis has been confirmed by Ceraudo et al. (34) who have shown that K17F activates ERK1/2 in a β-arrestin-dependent and G_i_ protein-dependent manner whereas K16P only activates G_i_ protein. This functional selectivity of apelin peptides indicates that the β-arrestin-dependent ERK1/2 activation and not the Gi-dependent signaling may participate in K17F-induced BP decrease. Indeed, when pE13A and K16P are injected intravenously in rats, they lost their capacity to decrease arterial blood pressure (BP) when compared with the corresponding natural peptides pE13F and K17F (20, 35).

The characterization of the internalization pattern of the ApelinR induced by apelin-36 or pE13F demonstrates that the internalized ApelinR/pE13F complex is rapidly recycled to the cell surface *via* a Rab4 pathway. The internalized ApelinR/apelin-36 complex is targeted to lysosomes through a Rab7 pathway. Both of these pathways are linked to β-arrestin 1 association with differences in spatio-temporal association (36). These differences are in agreement with studies showing that apelin-36 leads to a strong and sustained desensitization whereas the pE13F-induced desensitization is transient (21). Therefore, subtle differences exist between the apelin isoforms regarding their pharmacological properties, which may influence their physiological actions.

As numerous GPCRs, ApelinR may also form heterodimers *in vitro* with other GPCRs. ApelinR has been shown to dimerize with AT1, leading to an inhibition of Ang-II signaling by apelin (37–39). ApelinR may also heterodimerize with κ-opioid receptor, leading to an increase in cell proliferation through an increase of PKC and a decrease of protein kinase A (PKA) activity (40). In HUVEC cells, ApelinR has been shown to heterodimerize with bradykinin type 1 receptor, leading to an increase in cell proliferation and in phosphorylation of eNOs through a G_q_ protein-dependent PKC signaling pathway (41).

## Development of Metabolically Stable Apelin Analogs

The *in vivo* transient effects of apelin fragments suggested that endogenous apelin peptides have a short half-life. In addition, Gerbier et al. (33) showed that K17F and pE13F have a half-life in mouse plasma of 4.6 and 7.2 min, respectively, and Murza et al. (42) showed that pE13F has a 14 min half-life in rat plasma. Regarding apelin-36, Japp et al. (43) suggested from experiments conducted in healthy human subjects that the half-life of apelin 36 is lower than 5 min. This short half-life could be attributed to the action of metabolic enzymes including exo- and endo-peptidases.

The short *in vivo* half-life of apelin encouraged the development of metabolically stable apelin analogs for potential therapeutic applications.

Numerous technologies, such as PEGylation (44, 45), palmytoylation (46) conjugation to albumin, N-terminal acetylation (33), C-terminal amidation (47), use of unnatural amino acids (15, 33, 42, 48), or main chain modifications (cyclization) (49–51) have now been set up to increase the *in vivo* plasma half-life of peptides (52). Table 1 summarizes the pharmacological characteristics of the main metabolically stable apelin analogs described in this section.

**Table 1:** Metabolically stable apelin analogs.

	Reference	Affinity (*K*_i_, nM)	cAMP production inhibition (IC_50_, nM)	Half-life in plasma^a^ (min)
***Apelin-13***				
***pE-R-P-R-L-S-H-K-G-P-M-P-F (pE13F)***	(33)	***0.56*** ± ***0.07***	***1.68*** ± ***0.47***	***7.2***
E-R-P-R-L-S-H-K-G-P-Nle-P-2Nal	(48)	1.2 ± 0.1	20.5 ± 6	<120
E-R-P-R-L-S-H-K-G-P-Nle-P-4Br(F)	(48)	1.2 ± 0.1	12.4 ± 3	<60
E-R-P-R-L-S-H-K-G-P-Nle-Aib-F	(48)	1.8 ± 0.2	20.9 ± 7	<60
pE-R-P-R-L-S-H-K-G-P-Nle-P-Bpa	(42, 53)	0.38 ± 0.04	0.04 ± 0.02	55
pE-R-P-R-L-S-H-K-G-P-Nle-P-Y(O)Bn	(42, 53)	0.016 ± 0.002	0.35 ± 0.09	66
pE-R-P-R-L-S-H-K-G-P-Nle-P-F(L-αCH3)	(42, 53)	0.34 ± 0.02	0.07 ± 0.02	>120
Cyclo(1-6)C-R-P-R-L-C-H-K-G-P-M-P	(50)	300	2.7 ± 0.8	ND
Palmitoyl-E-R-P-R-L-S-H-K-G-P-Nle-Aib-F	(46)	ND	21.6 ± 4.5	1,740
Cyclo(7-11)pE-R-P-R-L-S-AllyG-K-G-P-AllyG-P-Y(O)Bn	(51)	1.7 ± 0.25	35 ± 11	ND
Ac-E-R-P-R-(D)L-S-Aib-K-(D)A-P-Nle-P-4Br(F)	(33)	2.11 ± 0.40	2.22 ± 1.00	86
pE-R-P-R-L-S-H-K-G-P-Nle-Aib-Br(F)	(15)	ND	ND	>60

***Apelin-17***				
***K-F-R-R-Q-R-P-R-L-S-H-K-G-P-M-P-F (K17F)***	(33)	***0.06*** ± ***0.01***	***0.30*** ± ***0.10***	***4.6***
Ac-K-F-(D)R-R-(D)Q-R-P-R-(D)L-S-Aib-K-(D)A-P-Nle-P-4Br(F) **(P92)**	(33)	0.09 ± 0.02	0.56 ± 0.32	24
CF3((CF2)7(CH2)2C(O)-K-F-R-R-Q-R-P-R-L-S-H-K-G-P-M-P-F **(LIT01-196)**	(33)	0.08 ± 0.01	1.71 ± 0.28	>1,440
K-F-R-R-Q-R-P-R-L-S-H-K-G-P-Nle-Aib-Br(F)	(15)	ND	ND	30

***Apelin-36***				
***L-V-Q-P-R-G-S-R-N-G-P-G-P-W-Q-G-G-R-R-K-F-R-R-Q-R-P-R-L-S-H-K-G-P-M-P-F***	(6, 7, 43)	***4.8*** ± ***0.24***	***0.52***	**<*5***^b^
40 kDa-PEG-Apelin-36	(44)	0.3	1.5	ND
Apelin-36-[L28C(30 kDa-PEG)]	(45)	ND	3,050 ± 2,100	ND

*^a^*Ex vivo* plasma half-life (unless otherwise specified)*.

*^b^In vivo plasma half-life*.

*ND, not determined; G, glycine; AllyG, allylglycine; P, proline; A, alanine; V, valine; L, leucine; I, isoleucine; M, methionine; C, cysteine; F, phenylalanine; Y, tyrosine; W, tryptophan; H, histidine; K, lysine; R, arginine; Q, glutamine; N, asparagine; E, glutamic acid; D, aspartic acid; S, serine; T, threonine; Aib, aminoisobutyric acid; Y(O)Bn, tyrosine(O)benryl; Bpa, benzoïlphenyl; Nle, norleucine; 2Nal, naphtalen-2-yl-propanoic acid; 4Br(F), 4-bromo-phenylalanine; PEG, polyethyleneglycol*.

*In bold and italic characters: native apelin peptides*.

*In bold characters: name of the analog as it is referred in the article*.

Most studies that aim to develop apelin analogs have focused on pE13F (15, 42, 46, 48, 50, 51, 53) or apelin-36 (44, 45); however, K17F, which had an affinity 10 times higher than that of pE13F for human ApelinR, was 10 times more efficient at inducing internalization of rat ApelinR and also decreased arterial BP more strongly than did pE13F (20).

Given these findings, metabolically stable K17F analogs were recently developed (15, 33). For this purpose, each amino acid of the sequence of K17F, which includes that of pE13F, was replaced with its D-isomer or with an unnatural amino acid. It was found in agreement with previous alanine scanning pE13F studies (17, 54) that replacement of the Arg^2^, Arg^4^, Ser^6^, Lys^8^, and Gly^9^ residues of pE13F with corresponding D-amino acids greatly decreased the ability of these compounds to bind to ApelinR or to inhibit FSK-induced cAMP production. Thus, combination of acetylation of Lys^1^ and the introduction of D-Arg^3^ and D-Gln^5^, D-Leu^9^, Aib^11^, D-Ala^13^, Nle^15^, and 4Br-Phe^17^ in K17F, generating P92, which had an affinity of 0.09 nM for the ApelinR, similar to that of K17F (0.06 nM). On the other hand, an original strategy for improving the protection of endogenous peptides against enzymatic degradation on the basis of the introduction of a fluorocarbon chain (FC) directly into the N-terminal part of K17F was used to generate LIT01-196. The presence of the FC on the apelin peptide had no impact on the affinity of LIT01-196 for ApelinR (*K*_i_ = 0.08 nM), or on its solubility in water (>10 mM).

Altogether these chemical modifications allowed to an extend the plasma half-lives of P92 by a factor of 6–11 and that of LIT01-196 by a factor of >100 relative to that of K17F. LIT01-196 displayed remarkable resistance to degradation by plasma enzymes, as >90% of the peptide remained unchanged after 24 h of incubation at 37°C. FC acylation of an endogenous peptide therefore seems to be an efficient way to extend its half-life in plasma.

P92 and LIT01-196 displayed full agonist activity for cAMP production, ERK1/2 phosphorylation (nanomolar range), induction of ApelinR internalization (subnanomolar range), and β-arrestin recruitment (33).

## Physiological Effects of Apelin

### Distribution of Apelin and Its Receptor

#### Brain

**Figure 3:**
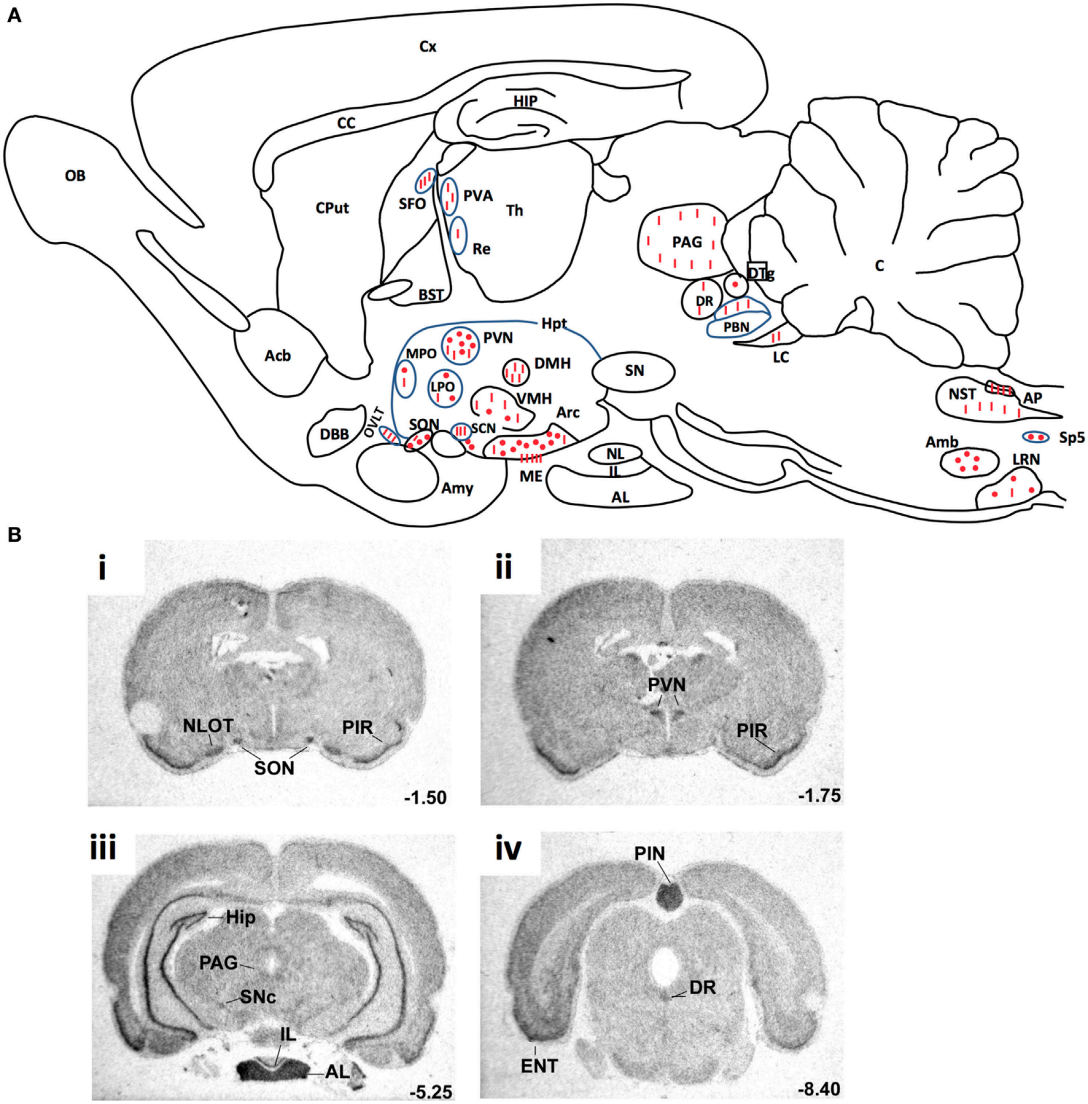
**(A)** Neuroanatomical distribution of apelin-IR cell bodies and nerve fibers on a parasagittal section of adult colchicine-treated rat brain. Apelin-IR cell bodies and nerve fibers are shown as dots and lines, respectively. Figure adapted from Ref. (56) with permission from the copyright holders. **(B)**. Distribution of the rat ApelinR mRNA expression in the adult rat brain. The figures were scanned directly from the X-ray film. Shown are representative frontal sections at anteriorities determined from the bregma indicated in the lower right corner. i–iv were hybridized with the antisense ApelinR cRNA probe. Scale bar: 2 mm. Figure adapted from Ref. (2) with permission from the copyright holders. Abbreviations: Acb, nucleus accumbens; Amb, nucleus ambiguus; Amy, amygdala; AL, anterior lobe of the pituitary gland; AP, area postrema; Arc, arcuate nucleus of the hypothalamus; BST, bed nucleus of the stria terminalis; C, cerebellum; CC, corpus callosum; Cput, caudate putamen; Cx, cerebral cortex; DBB, diagonal band of Broca; DMH, dorsomedial nucleus of the hypothalamus; DR, dorsal raphe nucleus; DTg, dorsal tegmental nucleus; ENT, entorhinal cortex; HIP, hippocampus; Hpt, hypothalamus; IL, intermediate lobe of the pituitary; LC, locus coeruleus; LPO, lateral preoptic area; LRN, lateral reticular nucleus; ME, median eminence; MPO, medial preoptic nucleus; NL, neural lobe of the pituitary; NLOT, nucleus of the lateral olfactory tract; NST, nucleus of the solitary tract; OB, olfactory bulb; OVLT, vascular organ of the lamina terminalis; PAG, periaqueductal gray; PBN, parabrachial nucleus; PIN, pineal gland; PIR, piriform cortex; PVA, paraventricular thalamic nucleus; PVN, paraventricular nucleus of the hypothalamus; Re, reuniens thalamic nucleus; SCN, suprachiasmatic nucleus; SFO, subfornical organ; SN, substantia nigra; SNc, pars compacta of the substantia nigra; SON, supraoptic nucleus; Sp5, spinal trigeminal nucleus; S, septum; Th, thalamus; VMH, ventromedial nucleus of the hypothalamus.

RT-PCR (8, 17), *in situ* hybridization (55) and *Northern blot* (4, 5) studies have shown that mRNAs coding for preproapelin and the ApelinR were distributed heterogeneously in different brain structures. The distribution of apelinergic neurons in the adult rat brain has been studied using a polyclonal antibody with a high affinity and selectivity for K17F, which also recognizes pE13F and apelin-36 (9, 32, 56). Apelin-immunoreactive (IR) neuronal cell bodies are abundant in brain structures that are involved in neuroendocrine control, food intake, drinking behavior, and regulation of BP, such as the hypothalamus and the medulla oblongata. They are particularly present in the supraoptic nucleus (SON), the magnocellular part of the paraventricular nucleus (PVN), the arcuate nucleus, the nucleus ambiguous, and the lateral reticular nucleus (56). Conversely, apelin-IR nerve fibers and nerve endings are more widely distributed in the brain: a high density was observed in the inner layer of the median eminence and in the posterior pituitary (32, 57). This suggests that, similarly to magnocellular vasopressinergic and oxytocinergic neurons, the apelinergic neurons of the PVN and the SON project into the posterior pituitary. This hypothesis was verified using double immunofluorescence staining showing that apelin was colocalized with arginine vasopressin (AVP) (9, 58) and oxytocin (57, 59) in magnocellular neurons. Apelin-IR cell bodies and fibers were also identified along the lamina terminalis, which is located in a region along the third ventricle and contains the subfornical organ (SFO), the organum vasculosum of the lamina terminalis (OVLT), and the median preoptic nucleus, which are involved in the control of drinking behavior (60, 61). The SFO and the OVLT, which contain fenestrated capillaries and no blood–brain barrier, constitute a link between peripheral events such as severe dehydration or hypovolemia and adaptive brain responses such as water intake or AVP release. Figure 3A shows the neuroanatomical distribution of apelin-IR cell bodies and nerve fibers on a parasagittal section of adult colchicine-treated rat brain.

The ApelinR is also widely distributed in the rat central nervous system Figure 3B (2, 4, 5). ApelinR mRNA has been identified using *in situ* hybridization in the piriform and the entorinal cortices, the hippocampus and in the pars compacta of the substantia nigra, the dorsal raphe nucleus and the locus coeruleus, which contain the monoaminergic neuronal cell bodies. The level of expression of ApelinR mRNA is high in the hypothalamic nuclei, including the SON and the PVN, the arcuate nucleus, the pineal gland, and the anterior and intermediate lobes of the pituitary gland (2). Moreover, double labeling studies combining *in situ* hybridization and immunohistochemistry have shown that, in the SON and PVN, mRNAs coding for the ApelinR (32, 62) as well as AVP receptors type 1a (V1a) and 1b (V1b), but not type 2 (V2R) (63), are coexpressed by magnocellular AVP neurons. This strongly strengthens the existence of an interaction between AVP and apelin.

#### Kidney

RT-PCR studies have shown that mRNA coding for preproapelin, as well as for ApelinR, is expressed in rat and human kidney (5, 17). Apelin-like immunoreactivity was also detected in human endothelial cells obtained from small intrarenal vessels (64). *In situ* hybridization studies in the rat kidney showed that ApelinR mRNA was expressed in the endothelial cells and vascular smooth muscle cells of the rat glomerular arterioles. ApelinR mRNA has also been detected and in all renal zones (26), most abundantly in the inner stripe (IS) and the outer stripe (OS) of the outer medulla (OM) (26). Furthermore, a high ApelinR mRNA expression was found in glomeruli and moderate in all the nephron segments including the collecting duct (CD) (26) where the V2R are also expressed (65).

### Maintenance of Water Balance by Apelin and Vasopressin through Central and Renal Effects

#### Central Effects of Apelin on AVP Neuronal Activity, AVP Release and Diuresis

**Figure 4:**
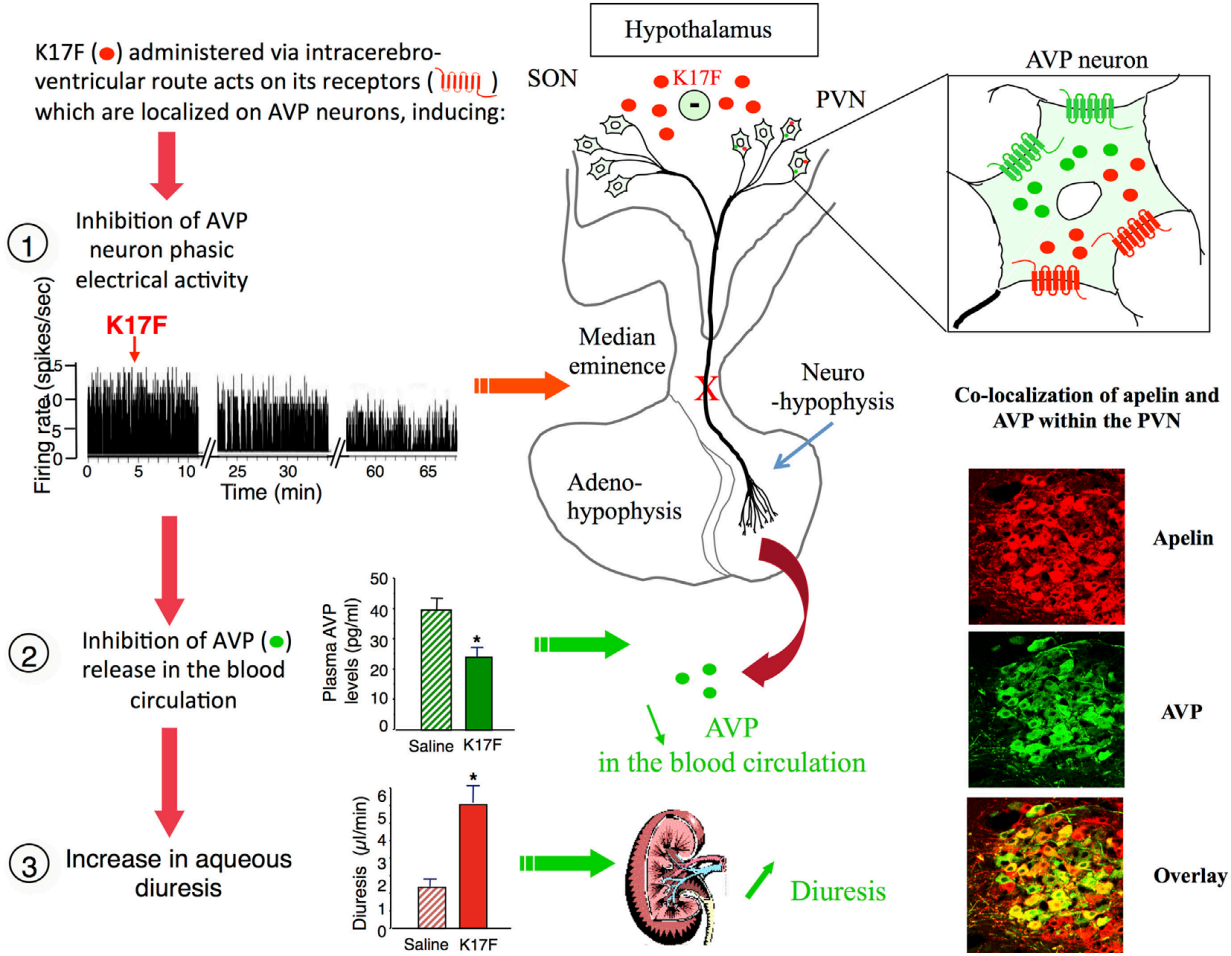
Central action of apelin on vasopressinergic neuron activity. Apelin and its receptor (ApelinR, red) are colocalized in magnocellular vasopressinergic neurons of the supraoptic nucleus and paraventricular nucleus (PVN), with arginine vasopressin (AVP) and vasopressin receptor type 1 (green). In lactating rats, i.c.v. injection of apelin 17 (K17F) inhibits the phasic electrical activity of AVP magnocellular neurons, leading to a decrease in AVP release in the bloodstream and an increase in aqueous diuresis. On the right of the figure are represented confocal images illustrating the distribution of apelin and AVP IR cell bodies within the rat PVN. A high colocalization of apelin (red) and AVP (green) has been detected in PVN magnocellular neurons. Dually labeled cells stand out in yellow when images are merged. Figure adapted from Ref. (11, 58) with permission from the copyright holders.

Arginine vasopressin, also known as anti-diuretic hormone, is a peptide that has antidiuretic and vasocontricting effects. It is synthesized and released by hypothalamic magnocellular AVP neurons in the blood circulation from the fenestrated capillaries of the posterior pituitary in response to variations in plasma osmolality and volemia (66, 67) or under the influence of neurohormones including natriuretic and angiotensin peptides (68, 69). The colocalization of AVP, apelin, V1, and ApelinR in magnocellular neurons suggests an interaction between apelin and AVP. This raises the possibility of an effect of apelin in response to osmotic or volemic stimuli. It has been hypothesized that independently from the feedback control exerted by AVP on its own release (69, 70), apelin may regulate AVP release. This hypothesis was checked in two animal models. First, the lactating rat, which exhibits an increase in the activity of magnocellular AVP neurons, leading to an increase in AVP synthesis and release in order to preserve water of the organism for maximal milk production (71, 72). In this model, the intracerebroventricular (i.c.v.) administration of apelin (K17F) (9) induces an inhibition of the phasic electrical activity of AVP neurons, reduces the release of AVP in the bloodstream and increases diuresis, without changes in sodium and potassium excretion (Figure 4). Second, in mice deprived of water for 24/48 h, a condition known to increase AVP neuron activity and systemic AVP release (73, 74), i.c.v. administration of K17F induced a significant decrease in systemic AVP release. These results suggest that apelin is probably released from the SON and PVN AVP cell bodies and inhibits AVP neuron activity and release through a direct action on the apelin autoreceptors expressed by AVP/apelin-containing neurons. This mechanism probably involves apelin acting as a natural inhibitor of the antidiuretic effect of AVP.

#### Effects of Apelin and Vasopressin on the Maintenance of Water Balance at the Kidney Level

In addition to a central action, the aquaretic effect of apelin probably involves a renal action because of the expression of ApelinR and preproapelin, as well as apelin immunoreactivity in the kidney (5, 17, 64). ApelinR mRNA has been detected in all renal zones, most abundantly in the IS of the OM (26). A high level of expression was also detected in the glomeruli and a moderate expression was observed in all nephron segments, especially in CD that express V2R In agreement with the presence of ApelinR mRNA in juxtamedullary efferent (EA) and afferent (AA) arterioles, application on glomerular arterioles precontracted by Ang-II of K17F, induced NO-dependent vasorelaxation by inhibiting the Ang-II induced rise in intracellular calcium mobilization (26). The apelin-dependent vasorelaxation observed in muscular EA, which gives rise to vasa recta, therefore strongly supports a key role of apelin in the control of renal medullary circulation (26).

On the other hand, it is well known that AVP by stimulating V2R in CD induces an increase in cAMP production and activates PKA which phosphorylates aquaporin type 2 (AQP2). This results in the insertion of AQP2 at the apical membrane of the principal cells of the CD (75, 76), leading to water reabsorption, decreasing diuresis, and plasma osmolality. The expression of ApelinR mRNA in the CD suggests that apelin could act as an aquaretic peptide through a direct action on this nephron segment. In agreement with this hypothesis, application of K17F on medullary CD inhibits cAMP production induced by dDAVP (a specific and selective V2R agonist) and decreased the dDAVP-induced calcium influx in CD cells (77). Finally, intravenous injection of K17F in increasing doses in lactating rats increases diuresis in a dose-dependent manner concomitantly with a significant decrease in urine osmolality and a decrease in AQP2 insertion at the apical membrane of CD (77), showing that K17F-induced diuresis is linked to a direct action of apelin on CD. Similarly, in other studies (78, 79), acute or chronic intravenous treatment with apelin-13 potently increased diuresis in male Sprague-Dawley rats.

Thus, the aquaretic effect of apelin is not only due to a central effect by inhibiting AVP release in the blood circulation but also to a direct action of apelin at the kidney level by increasing renal blood flow and counteracting the antidiuretic effect of AVP occurring via V2R in CD.

**Figure 5:**
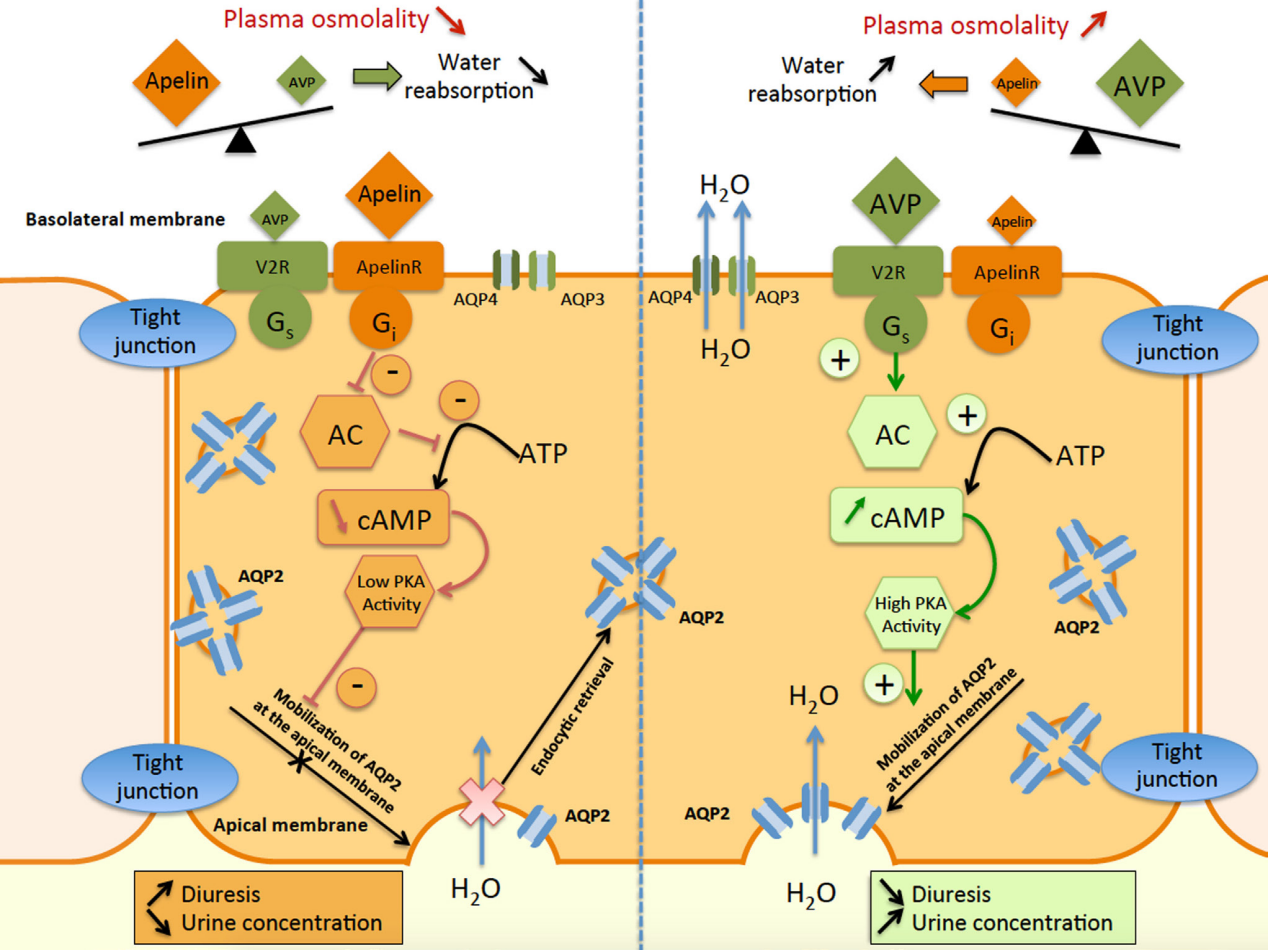
Schematic representation of apelin and vasopressin signaling pathways in principal cells of the collecting duct (CD). In the principal cells of the CD, arginine vasopressin binds to the V2-receptor (V2R), which stimulates adenylate cyclase (AC) *via* G_s_ coupling and increases cAMP production. This results in an increase in protein kinase A (PKA) activity, inducing the insertion of aquaporin type 2 (AQP2) at the apical membrane. Water reabsorption (H_2_O) is allowed by AQP2 at the apical membrane and AQP3 and AQP4 at the basolateral membrane, leading simultaneously to a decrease in diuresis and an increase in urine concentration. In contrast, apelin binds to the ApelinR, inhibiting AC through G_i_ coupling and decreasing cAMP production and PKA activity. This results in a decrease in AQP2 insertion at the apical membrane, leading to a decrease in water reabsorption and consequently in an increase in diuresis and a decrease in urine concentration.

These results also show that apelin and AVP have opposite effects on the CD and contribute to control plasma osmolality by regulating the reabsorption of water by the kidney (Figure 5).

Recent studies have underlined the role of apelin in controlling fluid homeostasis. As previously reported, water deprivation significantly decreases urine volume and increases urine osmolality in wild-type mice, whereas mice deficient for the ApelinR (ApelinR^−/−^) were unable to concentrate their urine to the same extent (80, 81). This lack of effect is likely not due to an inability of these mice to increase plasma AVP levels since similar increases in plasma AVP levels are seen in wild-type and ApelinR^−/−^ mice following water deprivation, suggesting the presence of an intact central vasopressinergic system in ApelinR^−/−^ mice. These observations have therefore led the authors to suggest that since the defect in water metabolism observed in ApelinR^−/−^ mice is not due to altered AVP neurosecretory function, it could be due to a defect in urine concentration at the kidney level. These results contrast with other studies published showing an aquaretic role of apelin in rodents. A possible explanation for this discrepancy is that in knock-out models, the total absence of ApelinRs during fetal and adult life could elicit compensatory mechanisms, leading to this opposite effects on urine output observed following an exogenous administration of apelin via i.c.v (9) and i.v. (26, 33, 77–79) routes. In addition, apelin gene expression in the brain has been also reported as a hydration-sensitive gene expression (82).

#### Effects of the Metabolically Stable Apelin Analogs, P92 and LIT01-196, at the Central and Kidney Levels

Central administration of P92 and LIT01-196 decreases dehydration-induced systemic AVP release in a dose-dependent manner and they are 6 and 160 times, respectively, more effective than K17F (33). These data suggest that P92 and LIT01-196, like K17F, rapidly reach the hypothalamic structures that are involved in AVP release from the posterior pituitary into the bloodstream after i.c.v. injection. They subsequently act on ApelinR expressed by AVP neurons to inhibit their phasic electrical activity, thereby preventing AVP release into the bloodstream. Furthermore, intravenous administration of P92 and LIT01-196 in anesthetized rats, at a dose in the nmol/kg range, potently increased urine output. In parallel, P92 and LIT01-196-induced vasorelaxation of rat glomerular arterioles, respectively, precontracted with Ang-II (33). This strongly suggests that these apelin analogs can act like K17F to increase medullary blood flow (26). Thus, by decreasing AVP release into the bloodstream and by increasing both renal blood flow and urine output, these new compounds should also have an aquaretic effect similar to that previously reported for K17F in lactating rats (9, 26). ApelinR agonists of this type would be particularly useful for the treatment of water retention and hyponatremia, making it possible to avoid the excessive sodium loss that is frequently reported in patients receiving thiazidic or loop diuretics.

#### Opposite Regulation of Vasopressin and Apelin following Water Deprivation

##### In Rodents

The colocalization and opposing actions of apelin and AVP on diuresis raise questions concerning how they are regulated to maintain body fluid homeostasis. To this end, the effect of water deprivation on the neuronal content and release of apelin and AVP were studied in rodents, and the effects of different states of hydration on plasma AVP and apelin levels were studied in human volunteers.

Following water deprivation in rodents, AVP is released into the blood circulation faster than it is synthesized, causing a depletion of AVP magnocellular neuronal stores (83). In parallel, water deprivation decreases plasma apelin levels and induces an increase in hypothalamic apelin neuronal content, especially in the PVN and SON AVP magnocellular neurons (9, 58). Following water deprivation, apelin therefore accumulates within AVP neurons rather than being released. This increase in neuronal apelin concentration observed in dehydrated rats is markedly reduced by the i.c.v. administration of a selective V1 receptor antagonist whereas the i.c.v. infusion of AVP has similar effects to dehydration on neuronal apelin content (58).

The apelin response to dehydration is therefore opposite to that of AVP (9, 83). These results imply that AVP and apelin are released separately by the AVP magnocellular neurons in which they are produced. In agreement with this hypothesis, double immunolabeling confocal microscopy studies show that a large proportion of apelin immunoreactivity colocalizes with AVP in magnocellular neurons in the SON and the PVN (Figure 4), although each peptide is found within distinct subcellular compartments (9, 58).

These *in vivo* animal studies show that the cross-regulation of apelin and AVP following osmotic stimuli has a physiologic purpose, allowing the maintenance of the organism water balance by preventing water excretion by the kidney after water deprivation.

##### In Humans

Such cross-regulation of apelin and AVP following osmotic stimuli has also been studied in humans. The relationship between osmolality, apelin, and AVP in the plasma was investigated in healthy men (10) following 2 h of hypertonic saline infusion to increase plasma osmolality or 30 min of oral water loading to decrease plasma osmolality.

Increased plasma osmolality simultaneously raised plasma AVP levels and decreased plasma apelin levels. Conversely, decreased plasma osmolality reduced plasma AVP levels and rapidly increased plasma apelin levels (10). These observations are consistent with plasma osmolality being a major physiologic regulator of plasma apelin levels in humans. Furthermore, the inverse relationship between the regulation of apelin and AVP is consistent with the findings in animal models following water deprivation. This strongly suggests that, like AVP, apelin helps to maintain body fluid homeostasis in humans and rodents.

#### Effects of Aging on the Cross Regulation of Apelin and Vasopressin

It has been shown in humans that elderlies have a higher risk of dehydration, due to lower levels of water intake and excessive water loss (84, 85). Both apelin and AVP contribute to maintain water homeostasis, and their regulation has therefore been investigated during aging.

Aging has been associated with an increase in the size of the magnocellular AVP neuron cell bodies (86), nucleoli (87), and Golgi apparatus (88, 89) in aged humans, and numerous studies have shown higher plasma AVP levels in aged rodents (90–93). In a recent study conducted in adult (3 weeks of age) or aged (22 weeks of age) male Wistar rats (94), intracellular AVP mRNA levels in the SON were lower in aged rats whereas intracellular apelin mRNA levels in the SON were higher. The authors then investigated the effect of dehydration on plasma AVP and apelin levels and showed an impaired response to dehydration of aged rats on both plasma AVP levels, which did not increase significantly after dehydration in aged rats, as well as on plasma apelin levels, which did not decrease significantly after dehydration as previously shown in adult rats (9).

Thus, there may be an absence of additional up- and downregulation of AVP and apelin in response to a sustained osmotic challenge, as the neuronal system may already be functioning at maximal capacity. Microglial inflammation and overproduction of interleukins have been proposed to contribute to the observed modifications of central apelin and AVP responses to osmotic changes in aged rats. The age-dependent impairment of the AVP/apelin response after an osmotic challenge could explain the poor tolerance of older patients to dehydration, although these results are yet to be confirmed in clinical studies involving elderlies.

### Apelin and Vasopressin Balance in Different Pathologies

#### Hyponatremia

Hyponatremia, defined by a plasma sodium concentration below 135 mmol/l, is a common metabolic disorder associated with an increased risk in mortality in hospitalized patients. It is usually secondary to free water retention rather than insufficient sodium intake. Various conditions have been associated with hyponatremia, including drug adverse events (diuretics, antidepressants), chronic heart failure (CHF), chronic liver disease, and the syndrome of inappropriate antidiuretic hormone secretion (SIADH). Copeptin corresponds to the C-terminal part of the AVP precursor and is composed of 77 amino acids. Copeptin is released mole to mole with AVP from the posterior pituitary in the blood circulation and plasma copeptin levels are used as a surrogate marker for AVP release in humans (95, 96). Copeptin measurement alone has proven useful in hyponatremic disorders to differentiate patients with primary polydipsia (PP), who have low copeptin plasma concentration and low urine osmolality, from other causes of hyponatremia such as SIADH, but do not allow to differentiate SIADH from other causes hyponatremic disorders such as diuretic-induced hyponatremia, hypovolemic hyponatremia, and hypervolemic hyponatremia (97).

##### Syndrome of Inappropriate Antidiuretic Hormone (SIADH)

In SIADH, abnormal AVP osmoregulation is the primary defect (98). A recent study showed that copeptin was useful to characterize five different subtypes of SIADH, according to plasma copeptin concentration response to hypertonic saline infusion (99). Since an opposite relationship is observed in healthy subjects between plasma apelin and AVP concentrations following osmotic stimuli, the apelin response to the AVP osmoregulation defect in SIADH was investigated (100). Expected ranges of plasma apelin, copeptin levels, and plasma to copeptin ratio for a given plasma sodium concentration were established in healthy subjects. In patients with SIADH, the sex- and age-adjusted apelin and copeptin levels were higher than in healthy subjects, by 26 and 75%, respectively. However, during an acute osmotic challenge, plasma copeptin levels were higher and plasma apelin levels lower than expected from plasma sodium and the plasma apelin to copeptin ratio decreased exponentially with natremia. Apelin to copeptin ratios as a function of natremia were outside the 95% predicted physiological limits for 86% of SIADH patients.

This study showed that apelin levels and apelin to copeptin ratios were inappropriate to natremia in SIADH patients, suggesting that the increase in plasma apelin secretion cannot compensate for the higher levels of vasopressin release and may contribute to the corresponding water metabolism defect.

##### Heart Failure

In CHF, the decrease in cardiac output and in effective circulating volume results in activation of baroreceptors. These activate the sympathetic nervous system, the renin–angiotensin–aldosterone system, and the release of AVP in the bloodstream. The increase in plasma AVP concentration leads to water reabsorption by the kidney (101). Mild to moderate hyponatremia is present in approximately 10% of CHF patients (102). Hyponatremic patients with advanced CHF often exhibit abnormally elevated AVP plasma levels (103), and hyponatremia has been associated with reduced survival and increased complications in CHF (104). Similarly to SIADH, the apelin to copeptin balance was investigated in hyponatremic CHF patients (100). In this study, plasma copeptin levels were increased by 190% compared to healthy subjects in hyponatremic patients. Plasma apelin levels were also slightly increased (by 25%). The plasma apelin to copeptin ratio was lower in CHF patients than in controls, suggesting that the mild increase in apelin secretion could not counteract the major increase in AVP secretion, leading to abnormal water metabolism and hyponatremia (100).

#### Polyuria-Polydipsia

In response to an increase in plasma osmolality, i.e. in case of dehydration or of hypertonic saline infusion, it has been shown both in humans and in animals that AVP secretion is increased and apelin secretion is decreased, allowing the reabsorption of water by the kidney. Thirst is also stimulated by the increase in plasma osmolality. In human pathology, an increase in both fluid intake (polydipsia) and diuresis (hypotonic urine output) is characteristic of the polyuria-polydipsia syndrome (PPS). Three entities are comprised in the PPS: central (complete or partial), diabetes insipidus (DI), nephrogenic (complete or partial) DI, and PP. Central DI (CDI) is secondary to complete or partial brain AVP deficiency mirrored by low plasma copeptin levels. In most cases, thirst perception is intact, resulting in polyuria, polydipsia. Severe hyperosmolality only occurs in case of fluid deprivation. Nephrogenic DI (NDI) is secondary to renal insensitivity to AVP and can be acquired or hereditary, due to mutations of the V2R or of AQP2. PP is not due to a deficiency or resistance of AVP but rather results from an excessive fluid intake over an extended period of time, in the absence of plasma osmolality regulation disorder. PP results from an abnormal thirst mechanism or a psychiatric disorder (105). Plasma copeptin measurement before and after water deprivation can be used to establish the differential diagnosis between each entity with good sensitivity and specificity (106). In order to determine if, similarly to what was observed in hypoosmolar disorders, apelin secretion was also impaired in PPS, the plasma apelin and copeptin levels were measured in PPS patients with complete CDI, complete NDI and PP(107). In patients with NDI, plasma copeptin levels were higher than those measured in healthy volunteers and plasma apelin levels were also increased by 61% compared to control values, probably in an effort of the apelin/AVP system to reestablish the water balance mediated by the two hormones. However, since in NDI AVP is unable to exert its antidiuretic effect at the kidney level, it is likely that only apelin exerts its deleterious effect, which results in polyuria.

In CDI, plasma copeptin levels were decreased by 52% compared to healthy volunteers whereas plasma apelin levels were only decreased by 18%. However, the plasma apelin to copeptin ratio was higher in CDI patients than in healthy volunteers. Possibly, the decrease in plasma apelin levels of patients with CDI is insufficient as compared to the decrease of AVP/copeptin to reestablish a balance between the antidiuretic effect of AVP and the diuretic effect of apelin, resulting in polyuria.

Patients with PP had normal plasma copeptin and slightly but significantly lower plasma apelin levels compared to healthy volunteers. However, the apelin to copeptin ratio was similar to that measured in healthy volunteers. This suggests that the normal apelin to copeptin ratio attests for balanced water homeostasis, whereas plasma apelin to copeptin ratio in CDI or NDI is increased or decreased compared to healthy volunteers, reflecting a disturbed water homeostasis.

Another interesting explanation is that apelin itself might also be involved directly in the pathogenesis of PP. Indeed, the decrease in plasma apelin levels in PP patients could account for altered drinking behavior (leading to an inadequate fluid intake in these patients) since i.c.v. injection of apelin into water deprived rats decreases drinking behavior (32). Further studies need to be conducted in order to determine if the use of the apelin to copeptin ratio could be a useful diagnosis tool in hyperosmolar disorders.

## Conclusion and Perspectives

The discovery of apelin as the endogenous ligand of the ApelinR is an important step in basic research and has clinical implications. In animal models, experimental data demonstrate that central injection of apelin into lactating rats inhibits the phasic electrical activity of AVP neurons, reduces plasma AVP levels, and increases aqueous diuresis. In the kidney, apelin increases diuresis by increasing renal blood flow and by counteracting the antidiuretic effect of AVP at the tubular level. Moreover, following water deprivation or salt loading, in humans and in rodents, AVP and apelin are conversely regulated to facilitate systemic AVP release and to avoid additional water loss at the kidney level. These data show that, together with AVP, apelin play a crucial role in the maintenance of body fluid homeostasis. In addition, in SIADH or CHF patients with hyponatremia, the apelin to copeptin balance is altered contributing to the water metabolism defect. The administration of apelin agonists in these patients could induce aqueous diuresis and therefore increase water excretion, reversing hyponatremia.

To date, there are no published preclinical or clinical studies utilizing ApelinR agonists/analogs in pathological animal models or patients with water metabolism disorders. The development of metabolically stable apelin analogs that exert longer-lasting and more potent effects than the endogenous peptide on aqueous diuresis and which additionally could only target the G_i_ signaling pathway like the endogenous apelin fragment K16P (biased agonist), represent promising candidates for the treatment of water retention/hyponatremic disorders.

## Author Notes

All appropriate permissions have been obtained from the copyright holders of Figures 1–4 that were adapted and reproduced for this manuscript.

## Author Contributions

All authors listed have made substantial, direct, and intellectual contribution to the work and approved it for publication.

## Conflict of Interest Statement

The authors declare that the research was conducted in the absence of any commercial or financial relationships that could be construed as a potential conflict of interest.
